# A systematic review on the effectiveness of robot-assisted minimally invasive gastrectomy

**DOI:** 10.1007/s10120-024-01534-1

**Published:** 2024-07-11

**Authors:** L. Triemstra, R. B. den Boer, M. M. Rovers, C. E. V. B. Hazenberg, R. van Hillegersberg, J. P. C. Grutters, J. P. Ruurda

**Affiliations:** 1 Department of Surgery, University Medical Center Utrecht, G04.228, 3508 GA Utrecht, The Netherlands; 2 Department of Medical Imaging, Radboud University Medical Center, Nijmegen, The Netherlands; 3 Department of Vascular Surgery, University Medical Center Utrecht, Utrecht, The Netherlands; 4 Department for Health Evidence, Radboudumc University Medical Center, Nijmegen, The Netherlands

**Keywords:** Gastric Cancer, Robot-assisted gastrectomy, RAMIG, IDEAL-framework, Implementation

## Abstract

**Background:**

Robot-assisted minimally invasive gastrectomy (RAMIG) is increasingly used as a surgical approach for gastric cancer. This study assessed the effectiveness of RAMIG and studied which stages of the IDEAL-framework (1 = Idea, 2A = Development, 2B = Exploration, 3 = Assessment, 4 = Long-term follow-up) were followed.

**Methods:**

The Cochrane Library, Embase, Pubmed, and Web of Science were searched for studies on RAMIG up to January 2023. Data collection included the IDEAL-stage, demographics, number of participants, and study design. For randomized controlled trials (RCTs) and long-term studies, data on intra-, postoperative, and oncologic outcomes, survival, and costs of RAMIG were collected and summarized.

**Results:**

Of the 114 included studies, none reported the IDEAL-stage. After full-text reading, 18 (16%) studies were considered IDEAL-2A, 75 (66%) IDEAL-2B, 4 (4%) IDEAL-3, and 17 (15%) IDEAL-4. The IDEAL-stages were followed sequentially (2A-4), with IDEAL-2A studies still ongoing. IDEAL-3 RCTs showed lower overall complications (8.5–9.2% RAMIG versus 17.6–19.3% laparoscopic total/subtotal gastrectomy), equal 30-day mortality (0%), and equal length of hospital stay for RAMIG (mean 5.7–8.5 days RAMIG versus 6.4–8.2 days open/laparoscopic total/subtotal gastrectomy). Lymph node yield was similar across techniques, but RAMIG incurred significantly higher costs than laparoscopic total/subtotal gastrectomy ($13,423–15,262 versus $10,165–10,945). IDEAL-4 studies showed similar or improved overall/disease-free survival for RAMIG.

**Conclusion:**

During worldwide RAMIG implementation, the IDEAL-framework was followed in sequential order. IDEAL-3 and 4 long-term studies showed that RAMIG is similar or even better to conventional surgery in terms of hospital stay, lymph node yield, and overall/disease-free survival. In addition, RAMIG showed reduced postoperative complication rates, despite higher costs.

**Supplementary Information:**

The online version contains supplementary material available at 10.1007/s10120-024-01534-1.

## Introduction

Globally, gastric cancer is the third leading cause of cancer-related mortality [1]. Open gastrectomy has been the traditional surgical approach for decades, either combined with peri-operative chemotherapy or not [2–4]. Laparoscopic gastrectomy was first introduced in 1994 to reduce surgical trauma through small incisions, resulting in less morbidity, shorter hospital stay, and improved cosmetic outcome [5]. Since then, the routine use of minimally invasive gastrectomy (MIG) has rapidly gained acceptance over the years [5–7]. Robot-assisted minimally invasive gastrectomy (RAMIG) was first described by Hashizume et al. in 2002 to overcome technical drawbacks such as limited range of motion and uncomfortable surgical positioning of conventional MIG [8]. Previous systematic reviews and meta-analyses on the safety and efficacy of RAMIG concluded that RAMIG provides favorable or comparable short-term outcomes to conventional laparoscopic gastrectomy (LG) or open gastrectomy (OG) in cardia and non-cardia gastric cancer patients, including less intraoperative blood loss, shorter hospital stays, and fewer postoperative complications [9–12]. In addition, similar or improved oncologic results such as total lymph node yield, radicality of resection, and mortality rates were reported.

Although RAMIG is gaining popularity, little is known about how it has been evaluated during its implementation into clinical practice. The evaluation of surgical procedures is complicated by factors related to the complexity of the surgical procedures, and surgeon-related factors including learning curve differences, and variability between hospitals [13]. In addition, surgical techniques are constantly evolving and subject to change even after their broad implementation in clinical practice. As a result, it is difficult to determine the appropriate timing for evaluating surgical procedures with well-designed and conducted randomized controlled trials (RCTs) [9].

The idea, development, exploration, assessment, and long-term follow up (IDEAL) framework was developed to describe the specific study designs and reporting standards to use at different stages of the implementation of surgical procedures and medical devices and to evaluate their introduction [14]. The IDEAL-stages range from stage 1 (first-in-human studies) to long-term follow-up of widely implemented techniques, stage 4. This framework aims to improve transparency, evaluation, and reporting of surgical innovations and medical devices to improve evidence-based practice [15]. This systematic review examined how RAMIG was evaluated during implementation into clinical practice based on the IDEAL-framework. Additionally, the current evidence for RAMIG was reviewed based on IDEAL-3 and 4 studies.

## Methods

This study protocol was prospectively registered in the online international PROSPERO database for systematic reviews under registration number CRD42022352208. This review was conducted in line with the Preferred Reporting Items for Systematic Reviews and Meta-Analyses (PRISMA) 2020 guidelines [16].

### Search strategy

A systematic literature search was undertaken in online databases including the Cochrane Library, Embase, Pubmed, and Web of Science for studies on RAMIG published up to January 2023. The search consisted of subject headings and text words, combining terms for *robotics* AND *gastrectomy* and synonyms. Table 1 provides the complete search strategy.

**Table 1:** Complete search strategy

Online database	Hits (n =)	Search
The Cochrane Library	127 hits	Robot*:ti,ab,kw OR telerobot*:ti,ab,kw AND gastrectom*:ti,ab,kw OR (gastric NEXT surger*):ti,ab,kw OR (gastric cancer NEXT surger*):ti,ab,kw OR (gastric NEXT resection*):ti,ab,kw OR RAMIG:ti,ab,kw
EMBASE*	238 hits	‘robotics’/exp OR ‘robot*’:ti,ab,kw OR ‘telerobot*’:ti,ab,kw AND 'gastrectomy'/exp OR 'gastrectom*':ti,ab,kw OR 'gastric surger*':ti,ab,kw OR 'gastric cancer surger*':ti,ab,kw OR 'gastric resection*':ti,ab,kw OR 'RAMIG':ti,ab,kw
Pubmed	839 hits	(Robotics[MeSH] OR robot*[Title/Abstract] OR telerobot*[Title/Abstract]) AND (Gastrectomy[MeSH] OR gastrectom*[Title/Abstract] OR gastric surger*[Title/Abstract] OR gastric cancer surger*[Title/Abstract] OR gastric resection*[Title/Abstract] OR RAMIG[Title/Abstract])
Web of Science	1134 hits	TS = (Robotic OR Robot* OR Telerobot*) AND TS = (gastrectom* OR gastric NEAR surger* OR gastric cancer NEAR surger* OR gastric NEAR resection* OR RAMIG)

*Filters: sources on MEDLINE, EMBASE and MEDLINE, publication types: article and article in press

### Study eligibility

Studies reporting on RAMIG compared to conventional techniques (laparoscopic or open approach) for cardia and non-cardia gastric cancer treatment were included. Only articles written in the English language and containing > 10 patients were eligible for inclusion in this systematic review. Reviews, study protocols, studies without available full texts, invited commentaries, and duplicates were excluded. In addition, we excluded non-comparative studies/robot-only studies, as we were interested in the reporting and evaluation of outcomes of RAMIG compared to conventional gastric cancer surgery.

### Study selection

Two researchers (LT and RdB) performed the title and abstract screening individually. In case of conflicts, disagreements were resolved through discussion to reach a consensus. The full-text screening was performed using the same approach, carried out by both researchers.

### Data extraction

Both researchers (LT and RdB) performed the data extraction of included studies. The following characteristics were included: IDEAL-stage, country of origin, year of publication, study design, number of included patients, and comparative approach (RAMIG versus laparoscopic and/or open total/subtotal gastrectomy). The IDEAL-stage of each included study was initially scored by LT and RdB using the flow diagram designed by the IDEAL collaboration (Table 2) [17]. Conflicts were resolved by a third and fourth researcher with extensive experience with the IDEAL-framework (JG and MR). If papers with different research aims from the same hospital were published at different times, only the paper with the majority of patients was used to calculate the total number of patients to avoid duplications. The results from both studies were incorporated in this systematic review. For IDEAL-3 and 4 studies we predefined a selection of outcomes, including extent of surgery, intra- and postoperative outcomes, oncological outcomes, overall/disease-free survival, overall quality of life, costs, surgical experience, and impact of learning curve (Supplementary Table 1).

**Table 2:** Summary of Idea, Exploration, Assessment, Long-term study and recommendations IDEAL-framework [17]

	1 Idea	2A Development	2B Evaluation	3 Assessment	4 Long-term study
Purpose	Proof of concept	Establish technical details and replicate early results	Learning	Assessment	Surveillance
Design	Structured case report	Prospective case series	Prospective comparative case series, feasibility RCT	RCT	Audit, registry, database
Number of patients	1	< 30	> 30	Guided by sample size calculation	Often large number
Inclusion criteria	Highly selected	Selected	Widening	Wide	Wide
Technical modifications	Report success and failures	Modifications allowed	Modifications allowed	No further modifications	No further modifications
Considered innovative procedure	Yes	Yes	Yes	No	No
Surgeon and center expertise	Details of pre-human work	Details of surgeon training	Details of mentoring and learning curve	Surgeons should be past the learning curve	Surgeons should be past the learning curve
Outcomes	Proof of concept; technical achievement, dramatic success, adverse events, surgeon view of the procedure	Mainly safety: technical and procedural success	Safety; clinical outcomes, short term patient centered outcomes, feasibility outcomes	Clinical outcomes, potential patient reported outcomes, health economic outcomes	Rare event, long term outcomes, quality assurance

### Data synthesis

Data were summarized in a narrative synthesis. Additionally, a schematic overview was produced for each country of origin, categorizing the included studies according to the different IDEAL-stages. In addition, over time, the corresponding IDEAL-stage of each included study was displayed graphically. No meta-analysis was performed, due to inter-study differences such as interval of postoperative complications (up to 30 days versus 90 days postoperatively), heterogeneity in treatment between patient groups (treatment with versus without neoadjuvant chemotherapy), and the variety in study designs (cohort studies versus RCT’s). Where feasible, comparisons were made between clinical outcomes based on the extent of gastrectomy (total or subtotal) and surgical approach (RAMIG, laparoscopic and/or open gastrectomy) in IDEAL-3 and 4 studies.

### Quality assessment

Risk of bias assessment of the included IDEAL-3 and 4 studies was performed by 1 researcher (LT). In case of uncertainty about the risk of bias, another researcher (RdB) was consulted to reach a consensus. The quality of the included IDEAL-3 and 4 studies was assessed according to the Cochrane Risk-of-Bias assessment tool version 2 (RoB 2 tool) for RCTs and ROBINS-I tool for non-randomized clinical trials (N-RCTS) [18, 19]. The RoB 2 tool assesses the validity of RCTs based on a risk of bias judgment in 5 specific domains: bias in the randomization process, deviation from intended interventions, missing outcome data, measurement of the outcome, and selective reporting. The risk of bias of each domain was judged as ‘low’, ‘high’, or ‘some concerns’. The ROBINS-E tool assesses the N-RCTs against 7 domains of bias for validity: confounding factors, measurement of exposure, participant selection, post-exposure interventions, missing data, outcome measurement, and selective reporting. The risk of bias for each domain was recorded as ‘low’, ‘some concerns’, ‘high’, or ‘very high’.

## Results

### Included studies

**Fig. 1:**
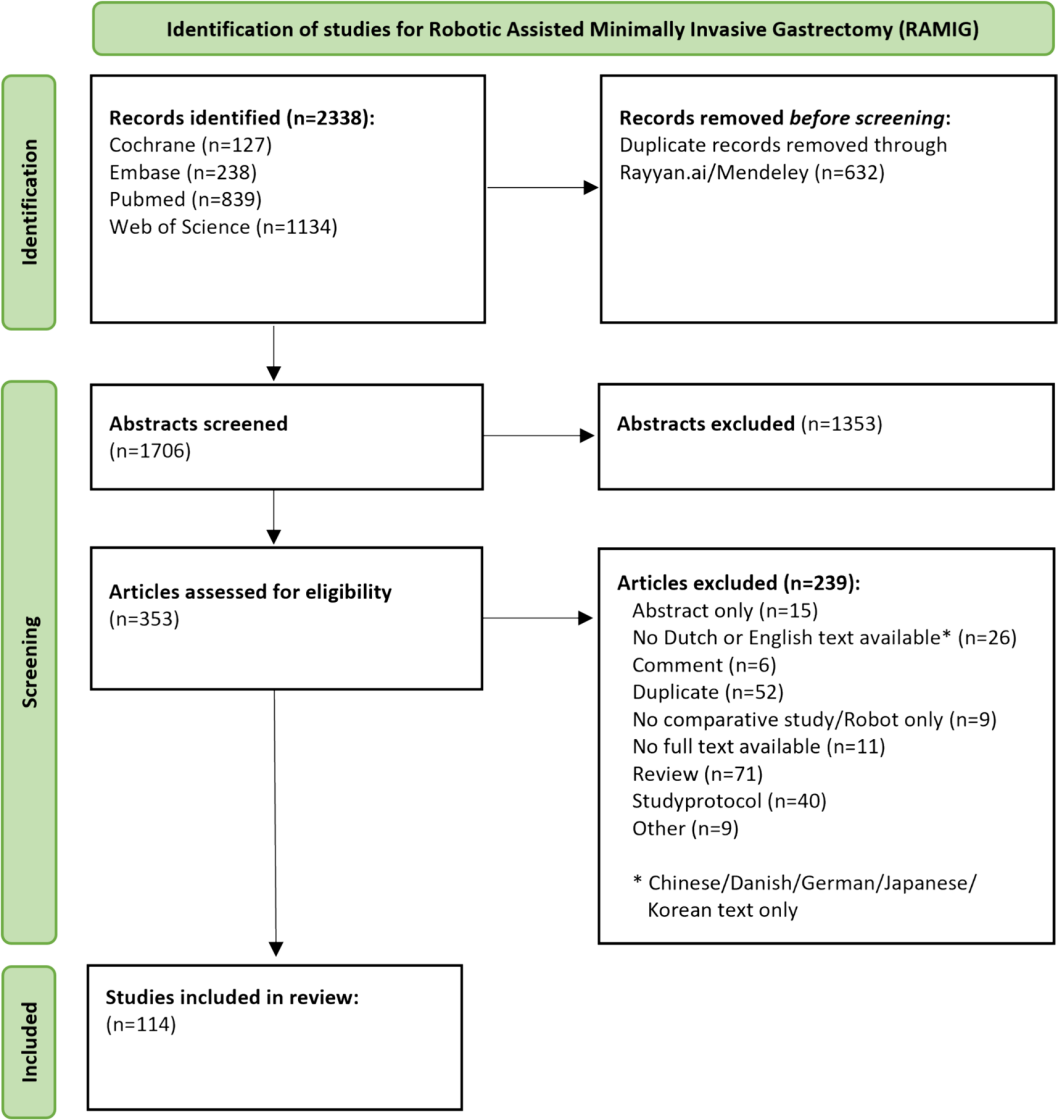
PRISMA Flowchart

In total, 2338 records were identified in the initial search. Following the removal of 632 duplicates, 1706 abstracts were screened. Ultimately, 114 studies were included in this study after screening 353 full texts (Fig. 1).

### General study characteristics and IDEAL-stage

**Fig. 2:**
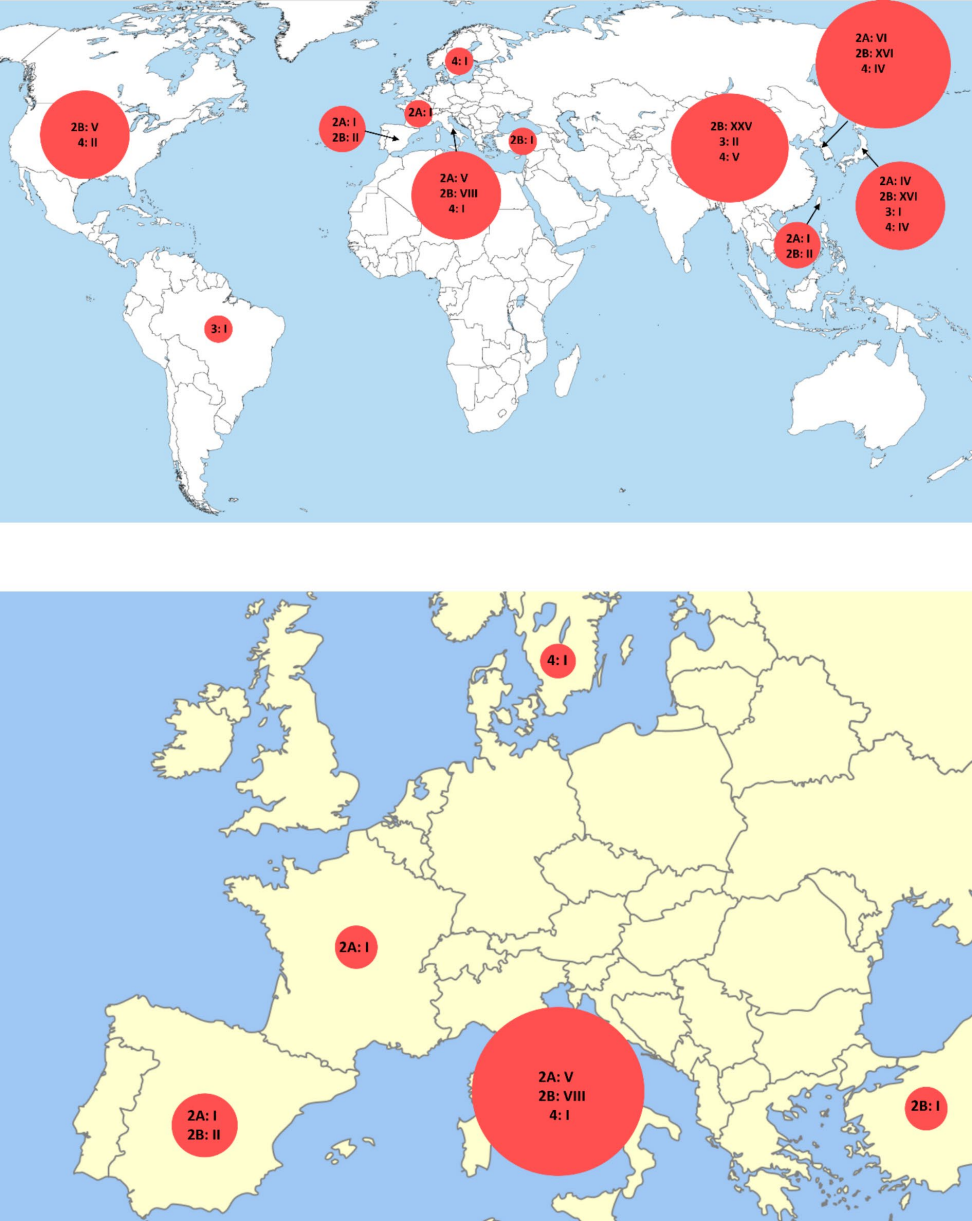
Distribution of published RAMIG studies according to IDEAL-2A, 2B, 3 and 4 stages. Upper = World, lower = Europe

**Fig. 3:**
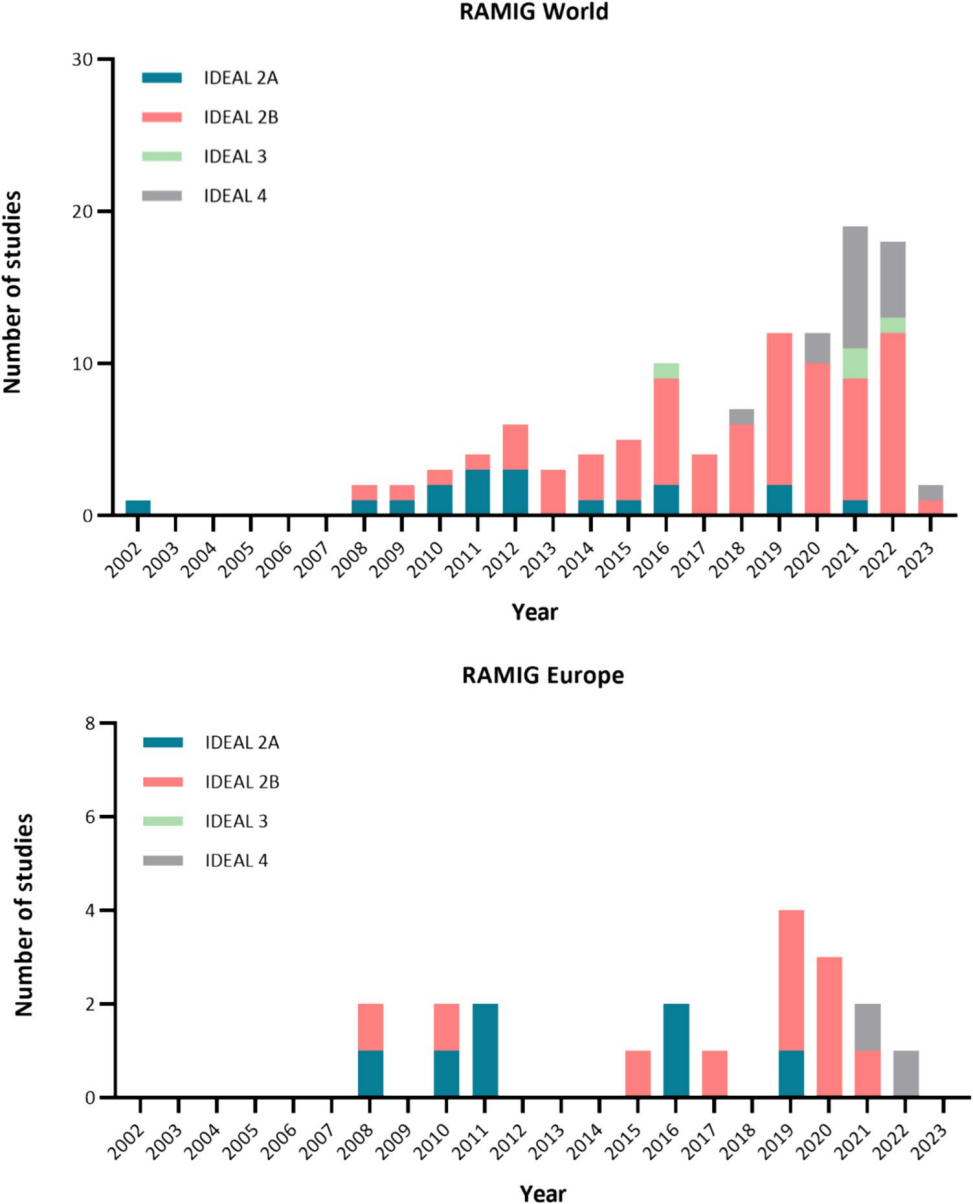
Course of IDEAL-stages 2A, 2B, 3, and 4 of included studies over time Worldwide (upper graph) and in Europe (lower graph)

None of the included studies reported an IDEAL-stage. In total 18 studies (16%) were classified as IDEAL-stage 2A, 75 studies (66%) as IDEAL-2B, 4 studies (4%) as IDEAL-3, and 17 studies (15%) as IDEAL-4. The first publication of early experiences with RAMIG dates from 2002 and originates from Japan (IDEAL-2A, Fig. 2 and 3)[8]. Six years later in 2008 the first publication in Italy followed (IDEAL-2A, Fig. 2) [20]. The results of the first IDEAL-2B study were published in 2009. In total 4 randomized controlled/clinical trials have been published (IDEAL-3), of which the first was published in 2016 and compared total/subtotal RAMIG with total/subtotal open gastrectomy (Fig. 3) [21–24]. Although IDEAL-3- and 4 long-term follow-up study results have been published, early IDEAL-2A studies are still being conducted in the same (Southeast Asia: Japan) or different (Europe: Italy, France, and Spain) continents (Fig. 3) [25–27]. Most of the included studies on RAMIG were conducted in China (n = 32, 39%), Korea (n = 26, 31%), Japan (n = 25, 30%), and Italy (n = 14, 12%).

### Study characteristics IDEAL 3- and 4 studies

Of the 114 included studies, n = 21 (18%) were classified as IDEAL-3 and 4 studies. These IDEAL-3- and 4 studies included in total 44.795 patients (median 700, range 60–30324 patients). Of these patients, 8.029 (18%) underwent RAMIG (n = 2.030 (5%) total RAMIG, n = 5.963 (13%) subtotal RAMIG, and n = 36 (0.1%) unknown), 14.319 (32%) underwent laparoscopic gastrectomy (n = 3.702 (8%) laparoscopic total gastrectomy, n = 10.514 (23%) laparoscopic subtotal gastrectomy, and n = 103 (0.2%) unknown), and 22.447 (50%) underwent open gastrectomy (n = 6.922 (15%) total open gastrectomy, n = 15.142 (34%) subtotal open gastrectomy, and n = 383 (1%) unknown). The study designs included retrospective cohort studies (n = 17), and RCTs (n = 4).

### Risk of *bias* within IDEAL-3 and 4 studies

**Fig. 4:**
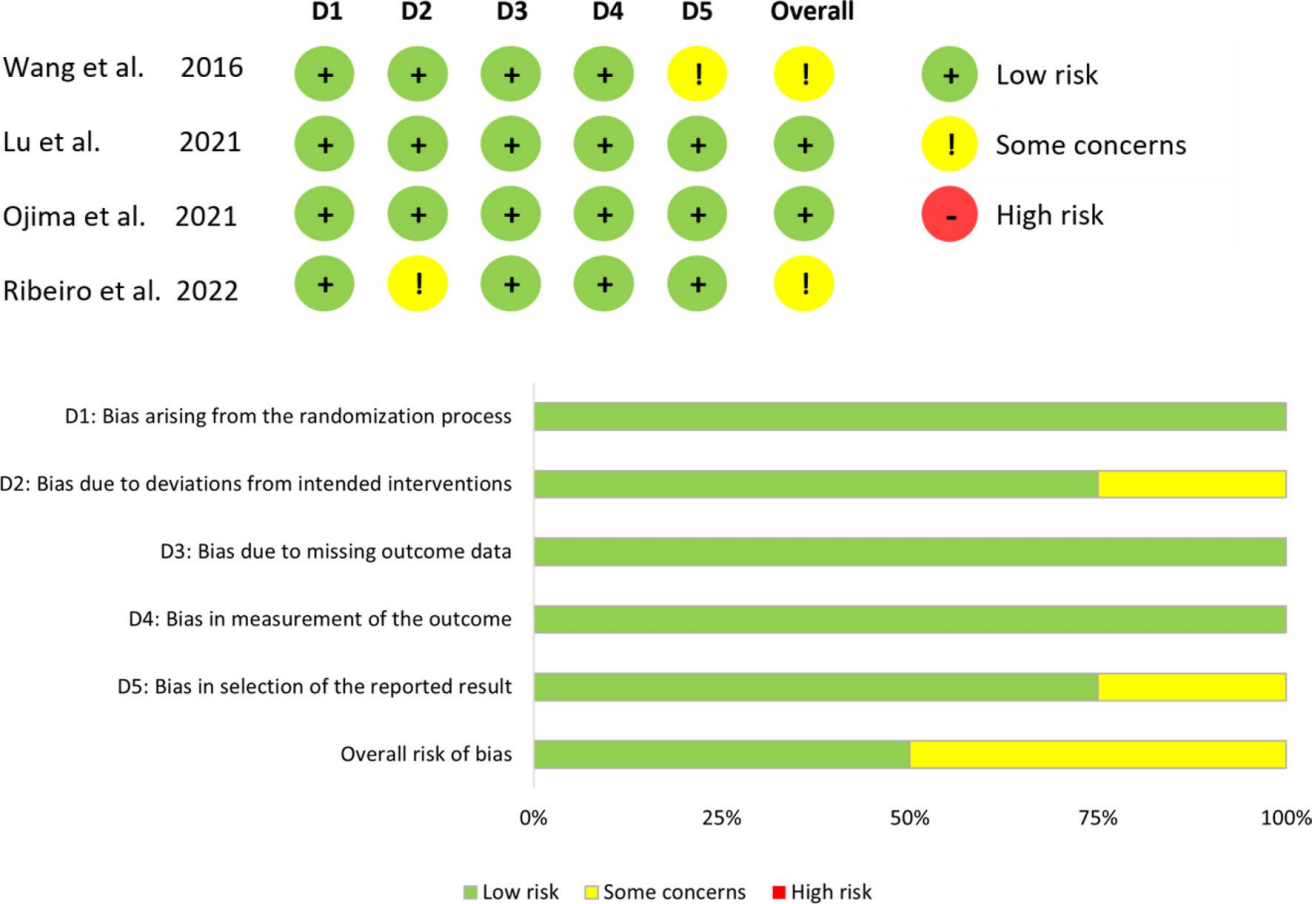
Summary of the risk of bias for randomized controlled/clinical studies [21–24]

**Fig. 5:**
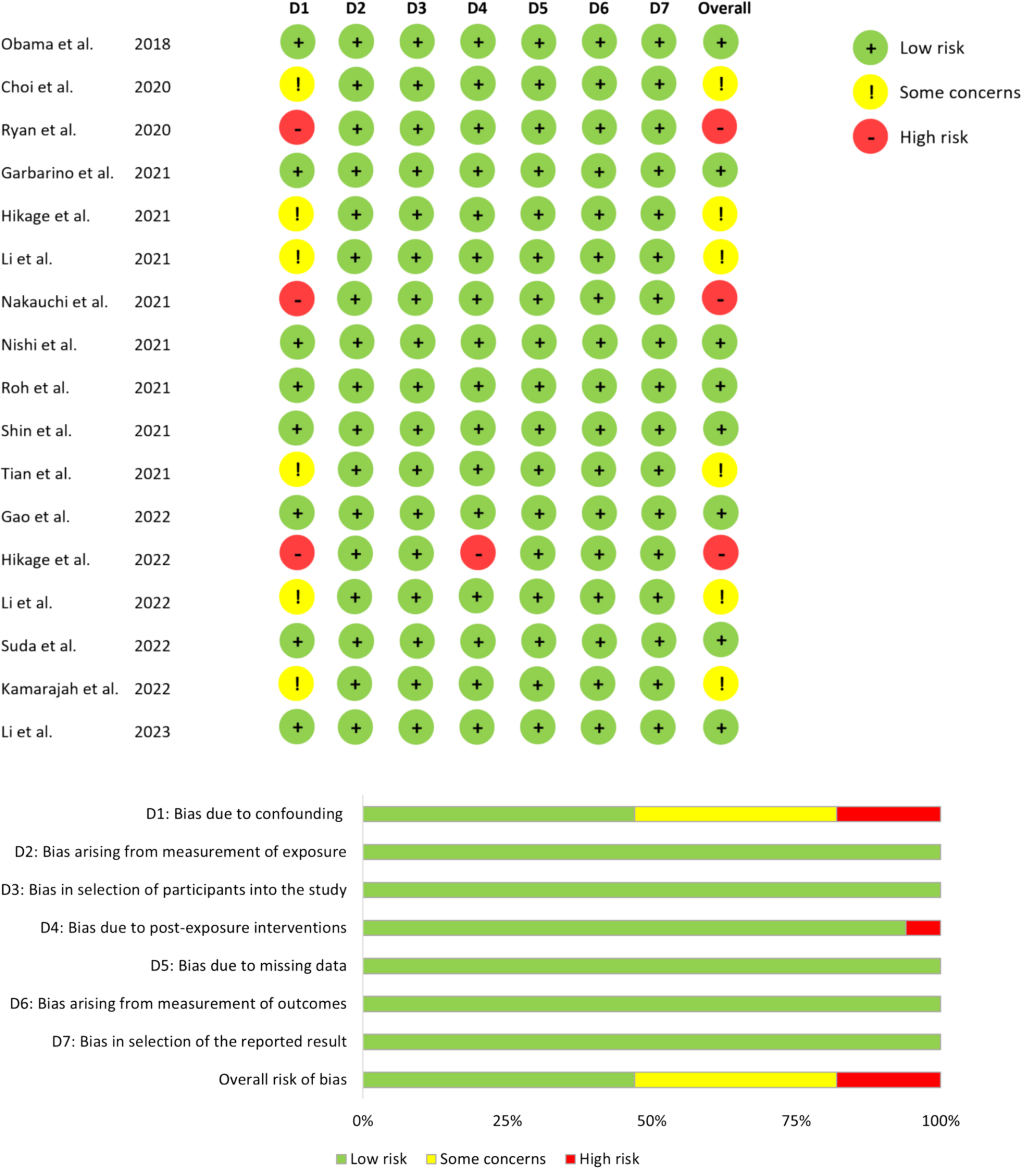
Summary of the risk of bias for non-randomized clinical trials [28–44]

Among the 4 RCTs, 2 (50%) showed a risk of bias with ‘some concerns’ for specific domains. In one study these concerns were related to a lack of a previously published study protocol and in another study concerns were caused by performing a per-protocol analysis instead of an intention-to-treat analysis excluding trial participants who did not receive their assigned intervention (Fig. 4) [21, 24]. In 17 N-RCTs, 6 (35%) showed a risk of bias with ‘some concerns’ regarding confounding bias. This was particularly the case in propensity-score matching studies, where important confounding factors such as comorbidities, neoadjuvant chemotherapy treatment, tumor size were not addressed, and/or information on data for which matching was performed was not reported in the baseline characteristics tables (Fig. 5) [28–33]. Three N-RCTS (18%) were at ‘high risk’ of bias due to baseline differences after PSM (n = 1) or no matching (n = 2) [34–36].

### Effectiveness of RAMIG according to IDEAL-3 studies

In total 4 RCTs on RAMIG were published [21–24]. A Brazilian (n = 65) and a Chinese (n = 311) single-center RCT compared total/subtotal RAMIG to open total/subtotal gastrectomy (OG), revealing similar complication rates (Clavien-Dindo (CD) grade I-V) (27.6% RAMIG versus 29.0% OG in the Brazilian RCT*,* and n = 14, 9.3% RAMIG versus n = 15, 10.3% OG in the Chinese RCT [21, 24]. Similar severe complication rates (CD ≥ IIIa or higher) (n = 4, 13.8% RAMIG versus n = 3, 9.7% OG, in the Brazilian RCT, and 28.6% RAMIG versus 33.4% OG, in the Chinese RCT) were reported. Similar lymph node yields (41.3 ± 15.1 RAMIG versus 42.4 ± 18.3 OG in the Brazilian RCT, and 30.1 ± 7.2 RAMIG versus 29.1 ± 6.7 OG in the Chinese RCT) were reported [21, 24]. A similar hospital stay in the Brazilian RCT (9.1 ± 5.5 days RAMIG versus 8.9 ± 5.6 days OG) was reported, in contrast to a shorter mean length of hospital stay in the Chinese RCT for RAMIG (5.7 ± 2.3 days RAMIG versus 6.4 ± 2.5 days OG). Furthermore, results on the R0-resection rates were only reported in the Brazilian RCT, which showed no difference (n = 29; 100% versus n = 29; 100%) [24].

A Chinese single-center RCT (n = 300) comparing robot-assisted distal gastrectomy (RDG) with laparoscopic distal gastrectomy (LDG) demonstrated reduced overall morbidity with RDG (n = 13, 9.2% RDG versus n = 25, 17.6% LDG) [22]. However, severe postoperative complications (CD ≥ IIIa) (n = 2, 1.4% RDG versus n = 2, 2.4% LDG), mean hospital stay (7.9 ± 3.4 days RDG versus 8.2 ± 2.5 days LDG), total lymph node yield (40.9 ± 11.2 RDG versus 39.9 ± 12.2 LDG), and 30-day postoperative mortality (0% versus 0%) were comparable. Cost differences between RDG and LDG were calculated, and divided into total and indirect costs. Total costs were defined as the costs during hospitalization and were calculated as the sum of direct and indirect costs. Direct costs included all items and costs of service in the care of the patient during hospitalization such as surgical equipment, laboratory tests, medicine, and so on. Indirect costs included the overhead cost of the building, the amortization of capital equipment and supplies, maintenance of services, utilities, and administrative staff. However, indirect cost was not further separated in the financial department. RDG incurred higher total and indirect costs (median $13,423 [$12,684–13,993] RDG versus $10,165 [$9,564–11,003] LDG, and median $4,835 [$4,568–5,092] RDG versus $780 [$602–970] LDG, respectively), yet lower direct costs when compared to LDG (median $8,683 [$8,081–9,071] RDG versus $9,300 [$8,858–9,9940] LDG).

Furthermore, a Japanese two-center RCT (n = 241) comparing total/subtotal RAMIG with total/subtotal LG reported reduced overall (CD ≥ II) and severe postoperative complications (CD ≥ IIIa) with RAMIG (n = 10; 8.5% versus n = 23; 19.3%, and n = 6; 5.1% versus n = 19; 16.0%) [23]. The conversion rate (n = 4; 3.4% versus n = 2; 1.7%), median hospital stay (12 versus 13 days), total lymph node yield (31.5 versus 35.0 lymph nodes), and R0-resection rates (97.4% versus 92.4%) did not differ statistically significant between RAMIG and LG (all *p*-values ≥ 0.05).

Each IDEAL-3 RCT reported information on the surgical experience and the impact of the learning curve in their centers (Supplementary Table 2). In the Eastern IDEAL-3 studies, surgeons performed a minimum of 20, 50, or 430 RAMIG cases, and > 40 or > 300 LG cases before participating in the RCT [21–23]. Furthermore, Ojima et al. stated that for a surgeon with extensive experience in open radical gastrectomies, the learning curve required no less than 30 cases for RAMIG [23]. The 4 surgeons participating in the Brazilian RCT were highly experienced in both OG and LG, certified as console surgeons in the Da Vinci platform, and standardized their technique using laboratory swine models [24]. This high-volume institution conducts over 100 surgical gastrectomies annually, and a qualified tutor was present during each performed RAMIG procedure.

#### Effectiveness of RAMIG according to IDEAL-4 studies

In total 13 of the 17 IDEAL-4 studies (76%) no significant differences were reported in terms of 1-, 3- and 5-year overall and disease-free survival rates between total/subtotal RAMIG versus total/subtotal LG or total/subtotal OG [29–31, 33, 34, 36–43]. In 3 of the 17 IDEAL-4 studies (18%) total/subtotal RAMIG showed prolonged 3-year (96.3% RAMIG versus 89.6% LG, and median 66.4 months RAMIG versus 63.6 months LG versus 42.5 months OG, respectively) and 5-year survival rates (96.5% (95%CI 89.5–98.9) RAMIG versus 90.1% (95%CI 85.3–93.4%) LG) in favor of RAMIG [32, 35, 44]. One study (6%) reported no survival outcomes between total/subtotal RAMIG and total/subtotal LG [28].

The nationwide database study by Kamarajah et al., which included 30.324 patients, compared rates of textbook outcomes and survival between RAMIG, LG, and OG. The results of this extensive database study indicated that RAMIG resulted in similar or improved 5-year survival rates compared to LG or OG (median 66.4 months RAMIG versus 63.6 months LG and 42.5 months OG, *p* = 0.800/*p* = 0.006), and similar complication rates (34% RAMIG versus 35% LG and 36% OG, p = 0.319) [32].

A Korean propensity score-matched cohort study showed no statistically significant differences in pre- and postoperative quality of life up to 3 years between total/subtotal RAMIG and total/subtotal LG, except better pre- and postoperative cognitive functioning scores for RAMIG versus LG [28]. The authors stated that this difference in cognitive functioning may have resulted from differences in baseline characteristics. Only 2 studies (12%) reported information on postoperative pain [28, 39]. A significantly lower postoperative pain score was found for total/subtotal RAMIG versus total/subtotal OG (0.95 ± 0.7 versus 1.24 ± 0.7, on the 4-point Verbal rating scale (VRS) [39]. Four studies (24%) showed significantly higher total costs for total/subtotal RAMIG compared to total/subtotal LG ($15,262 (no SD reported) RAMIG versus $10,945 LG, $13,607 ± $4,375 RAMIG versus $10,928 ± $3,918 LG, mean $13,608 ± $4,326 RDG versus $10,925 ± $3,925 LDG, and mean $14,185 ± $4,892 RAMIG versus $10,637 ± 4,398 LG, respectively) [30, 33, 42, 43]. The surgical experience and/or the impact of the learning curve were reported in 11 (65%) studies [28, 29, 31, 33, 35–38, 40, 43, 44] (Supplementary Table 3).

### Clinical implications and potential advantages according to extent of gastrectomy and surgical approach in IDEAL-3 and 4 studies

In total, 6 out of the 21 (29%) IDEAL-3 and 4 studies compared subtotal (n = 5) or total (n = 4) RAMIG with total/subtotal LG, either exclusively or via subgroup analysis [22, 30, 31, 37, 42, 43]. Gao et al. found no statistical significant differences between subtotal RAMIG and subtotal LG in overall complications (14% versus 17%, *p* = 0.242), or 3-year overall survival (76% versus 73%, *p* = 0.471), but found a statistical significant improvement in median lymph node yield (31.4 nodes versus 29.4, *p* = 0.015) for subtotal RAMIG [42]. Conversely, Lu et al. reported statistical significant less overall complications (9% versus 18%, *p* = 0.039) after subtotal RAMIG, with no difference in mean total lymph node yield (41 nodes versus 40, *p* = 0.452) [22]. Subgroup analyses comparing subtotal RAMIG and subtotal LG showed no differences in overall complications (11% versus 13%, *p* = 0.185), and (3-year) overall survival (85–86% versus 85–86%, both *p* > 0.05) [30, 31, 43]. Li et al. reported a significant improved mean lymph node yield (31.6 nodes versus 30.2, *p* = 0.002), and supraprancreatic lymph node yield (13.1 nodes versus 11.5, *p* < 0.001) after subtotal RAMIG [43]. Contrary, another study reported no differences in mean nodal yield (35.0 nodes subtotal RAMIG versus 36.9 subtotal LG, *p* = 0.138) [30].

Roh et al. found no statistically significant differences between total RAMIG and total LG in overall complications (31% versus 31%, *p* = 0.893), 3-year overall survival (99% versus 90%, *p* = 0.144), or mean lymph node yield (43.1 nodes versus 46.0, *p* = 0.232) [37]. Subgroup analyses comparing total RAMIG and total LG showed no differences in overall complications (17% versus 21%, *p* = 0.072), and (3-year) overall survival (77% versus 73–76%, both *p* > 0.05) [30, 31, 43]. Li et al. reported a significant improved mean lymph node yield (35.1 nodes versus 32.2, *p* = 0.001), and mean supraprancreatic lymph node yield (14.1 nodes versus 12.2, *p* < 0.001) after subtotal RAMIG. Contrary, another study reported no differences in mean lymph node yield (40.2 nodes total RAMIG versus 42.1 total LG, *p* = 0.288) [30].

## Discussion

This systematic review examined how the adoption of RAMIG into clinical practice has proceeded based on the IDEAL-framework. Furthermore, it summarized the current evidence base regarding RAMIG through IDEAL-3 and 4 long-term follow-up studies. It took 14 years after the description of the first RAMIG procedure until the results of the first RCT comparing total/subtotal RAMIG with open total/subtotal gastrectomy were published. During the implementation of RAMIG, the different stages of the IDEAL-framework were predominantly followed in sequential order (2A-2B-3-4). Although results of IDEAL-3 RCTs are available, IDEAL-2A studies are currently still ongoing. Moreover, results of the IDEAL-3 and 4 long-term follow-up studies included in this systematic review showed that total/subtotal RAMIG results in similar or improved outcomes compared to conventional surgery in terms of overall complications, hospitalization, lymph node yield, and overall- and disease-free survival.

This systematic review reveals that the different IDEAL-stages were followed mainly in chronological order during RAMIG implementation worldwide. However, the initial IDEAL-3 RCT results were published in China in 2016, while subsequent IDEAL-2A studies were conducted and published in France and Japan three years later [21, 43, 44]. Currently, both IDEAL-2B and long-term IDEAL-4 studies are being conducted simultaneously, both on the same and different continents [33, 39, 45, 46]. This publication sequence highlights the uneven progress of the IDEAL-stages worldwide, reflecting variations in the timing and implementation of RAMIG in various gastric cancer centers. The question arises regarding the necessity of strictly following each IDEAL-stage in chronological order when initiating RAMIG in a center, country, or continent. Given the ever-evolving nature of surgical techniques and the necessity for adaptability, it is inevitable that different IDEAL-stages in the field of RAMIG research are completed at varying times and in different continents, as observed in our findings. Remarkably, IDEAL-2B studies are repeatedly conducted worldwide, serving as a crucial precursor to advancing to an RCT or long-term follow-up study (IDEAL-3/4). IDEAL-2B assesses the safety and feasibility of performing RAMIG within a specific center based on short-term clinical outcomes and the impact of surgeons’ learning curves. The generalisability of results from high-volume centers in Asia to other continents with distinct presentations of cardia and non-cardia gastric cancer is questionable. Hence, it is reasonable that IDEAL-2B studies are carried out on various continents to guarantee the safety and skill advancement of surgeons in performing RAMIG.

Previous systematic reviews and meta-analyses have shown that total/subtotal RAMIG offers similar or improved short-term outcomes compared to total/subtotal OG and/or total/subtotal LG, including reduced blood loss, fewer postoperative complications, and shorter hospital stays [9–12]. These findings align with the IDEAL-4 long-term follow-up studies included in this systematic review. However, it is essential to exercise caution when interpreting these findings as the meta-analyses heavily relied on retrospective studies, leading to variations in clinical and methodological approaches across the included studies due to differences in study designs, potential confounding factors, and the risk of selection bias. In addition, some of the included studies did not specify the surgical methods for radical gastrectomy, and variety in surgical experience and proficiency of the robotic system in different surgeries existed [11]. Moreover, 9 (53%) IDEAL-4 studies included in this review raised ‘some concerns’ or showed a ‘high risk’ of bias due to unaddressed confounding factors [28–36].

To address these methodological limitations, conducting prospective, multicenter RCTs or alternative design IDEAL-3 studies seem the next step in the IDEAL-framework to impartially compare new surgical innovations with current standard surgical therapies and minimize the risk of bias [14, 15]. A key consideration is the feasibility and necessity of conducting ‘original’ RCTs to distinguish between two surgical innovations. Indeed, challenges in design and implementation emerge when conducting surgical RCTs, especially when both surgical techniques are already established as standard practice or when patient preferences for treatments hinder achieving the required number of participants within the allocated timeframe. According to a systematic review of the characteristics of RCTs in surgery, the sample sizes in most surgical RCTs are small and they focus mainly on minor clinical events [47]. Moreover, this systematic review revealed that most RCTs exhibited bias with some concerns (54.4%), a finding consistent with the 50% bias of some concerns in our study [21, 24, 47]. The previously mentioned challenges can, consequently, lead to a lack of statistical power in RCTs, hindering their ability to provide compelling evidence.

When comparing new surgical innovations to current standard surgical therapies, reporting of patient outcomes, including short- and long-term morbidity, mortality rates, oncologic outcomes, and quality-of-life outcomes, is crucial [14, 15]. Despite IDEAL-3 and 4 studies showing consistency in reported outcome measures, certain outcome measures like radicality of resection and quality-of-life outcomes, such as postoperative pain, were not routinely reported or investigated. This is in line with two previous studies comparing reporting standards for robot-assisted anti-reflux surgery and robot-assisted cholecystectomy, in which overall consistency in standardized reporting of outcomes was lacking [48, 49]. This heterogeneity in reported outcome measures makes the robust evaluation of different surgical techniques challenging among studies. Consequently, it is difficult to demonstrate any advantages or disadvantages of a new surgical technique, over conventional techniques. However, unlike other studies on reporting standards for other robotic surgical procedures, in the current study we found that the progression of the different IDEAL-stages and the associated reporting standards were in chronological order. This suggests that the IDEAL-framework was followed in the adoption of RAMIG, allowing for a comparison between RAMIG and conventional surgery.

Many studies outlined the potential technical benefits of robotic surgery, such as offering surgeons a three-dimensional high definition, tenfold magnified stable camera view, tremor suppression, improved ergonomics, and increased surgeon’s freedom of movement due to the articulated wrist instruments. The potential benefits to patients, and improved ergonomics for surgeons, form the theoretical basis for the superiority of RAMIG. However, limited data exist on the impact of RAMIG on patients’ quality of life and surgeons’ ergonomics. A report by the Netherlands Healthcare Institute, which focused on how to implement new surgical innovations in the future, pointed out that there is a divergence between the viewpoints presented in studies on robotic surgery for gastric cancer and the actual outcomes reported in practice [50]. The report emphasizes the need to involve all stakeholders in agreeing on the evaluation process and the outcome measures to be assessed before implementing new surgical innovations. These outcome measures should extend beyond traditional metrics like complications to include societal factors such as total cost. Additionally, ‘softer’ outcome measures like patient satisfaction, surgeon career satisfaction, and ergonomic benefits should also be considered. Moreover, surgeons who perform robot-assisted gastrectomies indicated that although they feel that this technique has added value, the measured ‘hard’ outcomes often do not demonstrate this added value in a statistically significant difference. In addition, the use of the robot during surgery may improve surgeon’s long-term sustainable employability, by preventing physical discomfort and fatigue, and work-related risk of musculoskeletal disorders related to laparoscopic surgery [51–53]. These shortcomings highlight the importance of standardization and adequate reporting of outcomes in studies comparing robotic surgery with conventional approaches, for example using a standardized set of outcomes, to enable transparent and robust conclusions to be drawn regarding the potential advantages or disadvantages of RAMIG [54].

The core outcome measures in effectiveness trials (COMET) Initiative develops Core Outcome Sets (COS) to standardize outcome measurements in clinical trials, benefiting medical decision-making and patients information [55, 56]. The RoboCOS study recently established a COS for robotic procedures involving various stakeholders [57]. This RoboCOS comprised 10 outcomes, including patient (treatment effectiveness, overall quality of life, disease-specific quality of life, complications including mortality), surgeon (precision/accuracy, visualization), organization (equipment failure, standardization of operative quality, cost-effectiveness), and population-level (equity of access) aspects. Future clinical trials on robotic surgery should adopt these outcomes to enable unbiased comparisons between interventions. The Upper GI International Robotic Association (UGIRA) is another initiative to facilitate the effective implementation and advancement of robotic gastric surgery worldwide and standardize robot-assisted gastric cancer surgery [58]. UGIRA developed a comprehensive international prospective registry for upper GI surgeons to enter intra- and postoperative outcomes of their RAMIG cases. This registry facilitates collaborative research on robotic gastric surgery to enhance surgical practices within the Upper GI community [59]. By integrating standardized outcome measures like RoboCOS, incorporating these outcomes into prospective databases such as UGIRA, and adhering to the reporting standards outlined by the IDEAL-framework throughout the various stages of development and implementation of new surgical techniques, transparent, reliable, and robust outcomes for procedures like RAMIG can be facilitated.

The economic viability of robotic surgery remains a subject of debate. Critics argue that the established benefits are insufficient, and the technique's high costs and longer operative times are concerning. Studies on the cost-effectiveness of RAMIG included in this systematic review show approximately 3000–5000 USD higher operation costs compared to conventional surgery, mainly due to robot acquisition, maintenance, and expensive disposable instruments [22, 30, 42–44, 60–65]. Interestingly, the Chinese RCT showed lower direct costs for RDG, and conversely higher indirect costs for RAMIG compared to LDG. However, the cause for the increased indirect costs for RDG could not be elucidated by the authors [22]. It is expected that these high costs will decrease in the future as more competitive robotic providers emerge. Additionally, the impact of robotic surgery on surgical career sustainability and patient quality-of-life, including the ability to return to work post-surgery, has not been factored into cost-effective analyses. The cost analysis of the ROBOT-trial, comparing robot-assisted esophagectomy (RAMIE) with open esophagectomy, showed that RAMIE resulted in fewer postoperative complications without increasing overall hospital costs [66]. Including these factors is crucial for a comprehensive evaluation of RAMIG’s cost-effectiveness and potential benefits at the patient, surgical, organizational, and population levels. The health economic model of Patel et al. could be used for this purpose to analyze whether the benefits of a robot-assisted procedure do compensate for the additional costs [67].

## Limitations and strengths

To the best of our knowledge, this is the first systematic review that comprehensively examined the worldwide implementation of RAMIG according to the different IDEAL-framework stages (2A-2B-3-4) and its current evidence base. However, there are several limitations associated to consider. First, since we only included comparative studies and excluded studies with less than 10 patients, outcomes of IDEAL-1 proof-of-concept studies were omitted. Second, some included studies predate the introduction of the IDEAL-framework in 2009, raising potential concerns about fairness in classifying these studies using the IDEAL-criteria over time. Third, although our analysis focused on the chronological order of publication years as an indicative of sequential progression through the IDEAL-stages, we recognize that this approach may not fully reflect the nuanced differences in study duration between small phase II-studies (IDEAL 2A/2B) compared to larger RCT’s (IDEAL-3). Factors such as study size, complexity, and resource availability influence this and should be taken into account when interpreting the timeline of RAMIG implementation in this systematic review. Fourth, the quality assessment was not performed following the PRISMA guidelines by two researchers, but by 1 researcher (LT). However, the second researcher (RdB) critically assessed the cases on which there was uncertainty about the risk of bias through the critical assessment of the first researcher (LT) to reach a consensus. Last, the exclusion of non-English articles may have inadvertently led to the omission of crucial studies, particularly those in Asian languages**.** These limitations should be considered when interpreting the findings and implications of this review.

## Future implications

Future research on robotic surgery for gastric cancer should prioritize examining long-term outcomes, quality of life, potential ergonomic benefits, and longevity of the surgeon and surgical staff with potential cost savings. Due to the lack of robust outcomes from a prospective multicenter RCT (IDEAL-3), a European RCT is currently being designed that will examine total lymph node yield, complications and long-term survival for RAMIG versus conventional LG in patients with locally advanced gastric carcinoma after neoadjuvant treatment. Additionally, over 1000 patients are currently being recruited for a Japanese multicenter phase-III RCT kown as the MONA LISA study, which aims to assess the superiority of RAMIG over LG for both early and avanced gastric cancer [68]. As robotic surgery is constantly evolving, the question remains whether every incremental change within robotic surgery requires going through every IDEAL-stage starting from IDEAL-1, or whether certain stages can be omitted. With future implementation of supporting algorithms in robotic devices, surgical quality and safety are expected to further improve. Moreover, robotic surgery and its technology can be applied as a tool to facilitate teaching highly complex procedures, such as gastrectomy, to future surgeons. This could potentially result in progressing through the learning curve faster, in a safe manner.

## Conclusions

During the implementation of RAMIG, the different stages of the IDEAL-framework were mainly followed in sequential order, although IDEAL-2A studies are still ongoing. IDEAL-3 and 4 long-term studies showed that total/subtotal RAMIG is similar or even better to conventional surgery in terms of postoperative recovery, oncological outcomes, and survival. In addition, total/subtotal RAMIG showed reduced postoperative complication rates, despite higher costs. However, evidence from large-scale prospective RCTs using standardized outcomes for potential benefits of RAMIG is currently lacking. To improve evidence transparency and robustness for future new robotic surgical procedures, utilizing the IDEAL-reporting guidelines and specific Robotic Core Outcome Sets (RoboCOS) is recommended.

### Supplementary Information

Below is the link to the electronic supplementary material.

Supplementary file1 (DOCX 125 KB)
