# Supplementary material for: A systematic review on the effectiveness of robot-assisted minimally invasive gastrectomy
Source: Gastric Cancer. 2024 Jul 11;27(5):932–46. doi: 10.1007/s10120-024-01534-1 (PMC11335791; doi:10.1007/s10120-024-01534-1)
Supplement: Supplementary file 1 — Supplementary file1 (DOCX 125 KB) [file 10120_2024_1534_MOESM1_ESM.docx]

**Supplementary Material**

**Supplementary Table 1.** Predefined selection of outcomes

| **Outcome measurements** | **Outcomes** |
| --- | --- |
| **Extent of surgery** | Total gastrectomy |
|  | Subtotal gastrectomy |
| **Survival** | Overall survival |
|  | Disease free survival |
|  | 30-day mortality* |
| **Intraoperative details** | Conversion to open (n; %) |
|  | Intraoperative complications (n; %) |
| **Postoperative outcomes** | Total complications (Clavien-Dindo-Classification (CDC) I-V) (n;%) |
|  | Severe complications (CD =/> IIIa) (n;%) |
|  | Postoperative pain score |
|  | Length of stay (days; median [IQR]/mean +/- SD) |
| **Oncological outcomes** | Lymph node yield (total noes; median [IQR]/mean +/- SD) |
|  | Radicality of resection (R0) |
| **Overall quality of life*** | PRO/PROM (quality of life questionnaires) |
| **Economic*** | Costs (total costs, indirect/direct costs etc.) |
| **Surgical experience** | Ergonomics |
| **Description of the impact of a learning curve? Give a short summary** | - |

* These outcome measures are also listed in the RoboCOS core outcome set

|  | **Outcomes** | **Wang et al. (2016)**[21]  **(n=311)** | | | **Lu et al. (2021)** [22]  **(n=300)** | | |
| --- | --- | --- | --- | --- | --- | --- | --- |
|  |  | **RG versus OG** | | | **RDG versus LDG** | | |
|  |  | **Robot**  **(n=158)**  **After exclusion (n=151)** | **Open**  **(n=153)**  **After exclusion (n=145)** | **Difference between the groups (95% CI)** | **Robot**  **(n=141)** | **Laparoscopic**  **(n=142)** | **Difference between the groups (95% CI)** |
| **Extent of surgery** | Total gastrectomy (n (%))  Subtotal gastrectomy (n (%)) | 47 (31%) 104 (69%) | 53 (37%) 92 (63%)_ | *p*=0.329 | 0 (0%) 141 (100%) | 0 (0%) 142 (100%) | NA |
| **Survival** | Overall survival | NA | NA | NA | NA | NA | NA |
|  | Disease free survival | NA | NA | NA | NA | NA | NA |
|  | 30-day mortality | 0 | 0 | 0 | 0 (0.0%) | 0 (0.0%) | NA |
| **Intraoperative details** | Conversion to open (n (%)) | NA | NA | NA | NA | NA | NA |
|  | Intraoperative complications (n (%)) | 0 | 0 | - | 5 (3.5%) | 9 (6.3%) | *p*=0.279 |
| **Clinical outcomes** | Total complications (CD I-V) (n (%)) | 14 (9.3%) | 15 (10.3%) | *p*=0.856 | **13 (9.2%)** | **25 (17.6%)** | ***p*=0.039** |
|  | Severe complications (CD =/> IIIa) (n (%)) | 4 (28.6%) | 5 (33.4%) | *p*=0.756 | 2 (1.4%) | 3 (2.1%) | - |
|  | Postoperative pain score | NA | NA | NA | NA | NA | NA |
|  | Length of stay (days; median [IQR]/mean +/- SD) | **5.7 +/- 2.3** | **6.4 +/- 2.5** | ***p*=0.021** | 7.9 +/- 3.4 | 8.2 +/- 2.5 | *p*=0.062 |
| **Oncological outcomes** | Lymph node yield | 30.1 +/- 7.2 | 29.1 +/- 6.7 | *p*=0.447 | 40.9 +/- 11.2 | 39.9 +/- 12.2 | *p*=0.452 |
|  | Radicality of resection (R0) | NA | NA | NA | NA | NA | NA |
| **Overall quality of life** | PRO/PROM (questionnaires) | NA | NA | NA | NA | NA | NA |
| **Economic** | Costs | NA | NA | NA | **Total cost:** median $13423 [$12684-13993]  **Indirect median cost:** $4835 [$4568-5092]  **Direct cost:** $8683 [$8081-9071] | **Total cost:** median $10165 [$9564-11003]  **Indirect median cost:** $780 [$602-970]  **Direct cost:** $9300 [$8858-9940] | ***p*<0.001**  ***p*<0.001**  ***p*<0.001** |
| **Surgical experience** | Ergonomics | NA | NA | NA | NA | NA | NA |
| **Impact learning curve included? If yes, give a short description** | | ‘According to our experience, for a surgeon with extensive experience in open radical gastrectomy, the learning curve required no less than 30 cases.’ | | | The Da Vinci Robotic system was used to perform all RGs by the same group of surgeons with experience of more than 300 laparoscopic and 50 robotic operations for GC before joining the trial. | | |

**Supplementary Table 2.** IDEAL-3 studies

RG = robot-assisted gastrectomy. OG = open gastrectomy. RDG = robot-assisted distal gastrectomy. LDG = laparoscopic distal gastrectomy. CD = Clavien-Dindo. SD = standard deviation. IQR = interquartile range.

|  | **Outcomes** | **Ojima et al. (2021)** [23] **(n=236)** | | | **Ribeiro et al. (2022)** [24] **(n=65)** | | | |
| --- | --- | --- | --- | --- | --- | --- | --- | --- |
|  |  | **RG versus LG** | | | **Randomized trial RG vs OG** | | | |
|  |  | **Robot**  **(n=117)** | **Laparoscopic**  **(n=119)** | **Difference between the groups (95% CI)** | **Robot**  **(n=33)**  **After exclusion (n=29)** | **Open**  **(n=32)**  **After exclusion (n=31)** | | **Difference between the groups (95% CI)** |
| **Extent of surgery** | Total gastrectomy (n (%))  Subtotal gastrectomy (n (%)) | 38 (32%)  79 (68%) | 24 (20%)  95 (80%) | *p*=0.080 | 5 (17%)  24 (83%) | 4 (13%)  27 (87%) | *p*=0.727 | |
| **Survival** | Overall survival | NA | NA | NA | NA | NA | NA | |
|  | Disease free survival | NA | NA | NA | NA | NA | NA | |
|  | 30-day mortality | 0 | 0 | *p*=0.990 | 0 | 0 | NA | |
| **Intraoperative details** | Conversion to open (n (%)) | 4 (3.4%) | 2 (1.7%) | *p*=0.440 | 2 (6.7%) | NA | NA | |
|  | Intraoperative complications (n (%)) | NA | NA | NA | 1 (3.4%) | 0 | NA | |
| **Clinical outcomes** | Total complications (CD I-V) (n (%)) | **10 (8.5%)** | **23 (19.3%)** | ***p*=0.020** | 8 (27.6%) | 9 (22.6%) | NA | |
|  | Severe complications (CD =/> IIIa) (n (%)) | **6 (5.1%)** | **19 (16.0%)** | ***p*=0.010** | NA | NA | NA | |
|  | Postoperative pain score | NA | NA | NA | NA | NA | NA | |
|  | Length of stay (days; median [IQR]/mean +/- SD) | 12 [7-43] | 13 [6-45] | *p*=0.990 | 9.1 +/- 5.5 7 mean (6-11) | 8.9 +/- 5.6 7 (5-10) | *p*=0.854 | |
| **Oncological outcomes** | Lymph node yield (median [IQR]) | **31.5 [10-103]** | **35 [5-129]** | ***p*=0.050** | 41.3 +/- 15.1 | 42.2 +/- 18.3 | *P*=0.805 | |
|  | Radicality of resection (R0) | 114 (97.4%) | 110 (92.4%) | *p*=0.100 | 29 (100%) | 29 (100%) | NA | |
| **Overall quality of life** | PRO/PROM (questionnaires) | NA | NA | NA | NA | NA | NA | |
| **Economic** | Costs | NA | NA | NA | NA | NA | NA | |
| **Surgical experience** | Ergonomics | NA | NA | NA | NA | NA | NA | |
| **Impact learning curve included? If yes, give a short description)** | | To prevent surgeon bias, participating surgeons satisfy the following criteria: experience of more than 40 LG, experience of more than 20 RG, qualified surgeon according to endoscopic surgical skill qualification system, Japan Society for Endoscopic Surgery, board certified fellow of the Japanese Society of Gastroenterological Surgery. LG is performed by 3 surgeons (TO, MiN, MaN), RG by 1 surgeon (TO), these doctors each fulfill requirements for participation, and have sufficient training in dry and wet labs. | | | The procedures were performed by 4 surgeons, all with extensive experience in both OG and LG. They were certified as console surgeons in the Da Vinci platform by Intuitive. Before the study started, the technique was standardized in laboratory with swine models. A qualified tutor was present at the operating room during the surgical treatment and the Da Vinci Si system was used. Considering Western standards, our institution has high volume, with >100 procedures for GC per year. | | | |

RG = robot-assisted gastrectomy. OG = open gastrectomy. LG = laparoscopic gastrectomy. RDG = robotic distal gastrectomy. LDG = laparoscopic distal gastrectomy. CD = Clavien-Dindo. SD = standard deviation. IQR = interquartile range.

**Supplementary Table 3.** IDEAL-4 studies

|  | **Outcomes** | **Obama et al. (2018)** [41]  **(n=840)** | | | |
| --- | --- | --- | --- | --- | --- |
|  |  | **RG versus LG** | | | |
|  |  | **Study type: PSM (1:1)** | | | |
|  |  | **Robot (n=311)** | **Laparoscopic (n=311)** | **Difference between the groups (95% CI)** | |
| **Extent of surgery** | Total gastrectomy (n (%))  Subtotal gastrectomy (n (%)) | 82 (27%)  229 (73%) | 78 (25%)  233 (75%) | *p*=0.783 | |
| **Survival** | Overall survival | N=283  93.2% (95%CI 89.8-95.5))  (5-years OS) | N=286  94.2% (95%CI 91.0-96.3) (5-years OS) | *p*=0.521 | |
|  | Disease free survival | N=274  90.7% (95%CI 86.9-93.4) (5-years DFS) | N=281  92.6% (95%CI 89.1-95.0) (5-years DS) | *p*=0.229 | |
|  | 30-day mortality | 0 (0.0%) | 0 (0.0%) | NA | |
| **Intraoperative details** | Conversion to open (n (%)) | 0 (0.0%) | 1 (0.3%) | *p*>0.999 | |
|  | Intraoperative complications (n (%)) | NA | NA | NA | |
| **Clinical outcomes** | Total complications (CD I-V) | NA | NA | NA | |
|  | Severe complications (CD =/> IIIa) | NA | NA | NA | |
|  | Postoperative pain score | NA | NA | NA | |
|  | Length of stay (days; median [IQR]/mean +/- SD) | 7.2 +/- 10.0 | 7.0 +/- 12.1 | *p*=0.806 | |
| **Oncological outcomes** | Lymph node yield  (median [IQR]) | 40.0 +/- 15.2 | 40.1 +/- 15.2 | *p*=0.901 | |
|  | Radicality of resection (R0) | NA | NA | NA | |
| **Overall quality of life** | PRO/PROM (questionnaires) | NA | NA | NA | |
| **Economic** | Costs | NA | NA | NA | |
| **Surgical experience** | Ergonomics | NA | NA | NA | |
| **Impact learning curve included? If yes, give a short description** | | No | | |  |
|  | |  |  |  |  |

RG = Robot-assisted gastrectomy. LG = Laparoscopic gastrectomy. CD = Clavien-Dindo. OS = overall survival. DFS = disease free survival. NA = not applicable. SD = standard deviation. PSM = propensity score matched. IQR = interquartile range.

|  | **Outcomes** | **Ryan et al. (2020)** [34]  **(n=1893)** | | | **Choi et al. (2020)** [28]  **(n=321)** | | |
| --- | --- | --- | --- | --- | --- | --- | --- |
|  |  | **RG versus LG** | | | **RG versus LG** | | |
|  |  | **Study type: PSM (1:2)** | | | **Study type: multicenter prospective cohort study, PSM (1:1)** | | |
|  |  | **Robot**  **(n=631)** | **Laparoscopic**  **(n=1262)** | **Difference between the groups (95% CI)** | **Robot**  **(n=131)** | **Laparoscopic**  **(n=131)** | **Difference between the groups (95% CI)** |
| **Extent of surgery** | Total gastrectomy (n (%))  Subtotal gastrectomy (n (%)) | 174 (28%)  457 (72%) | 352 (28%)  910 (72%) | *p*=0.885 | 22 (17%)  109 (83%) | 19 (15%)  112 (85%) | *p*=0.875 |
| **Survival** | Overall survival | 56.2 (median, in months for a period of 70 months) | 49.2 (median, in months for a period 0f 70 months) | *p*=0.405 | NA | NA | NA |
|  | Disease free survival | NA | NA | NA | NA | NA | NA |
|  | 30-day mortality | 19 (4.5%) | 26 (2.7%) | *p*=0.101 | NA | NA | NA |
| **Intraoperative details** | Conversion to open (n (%)) | NA | NA | NA | NA | NA | NA |
|  | Intraoperative complications (n (%)) | NA | NA | NA | NA | NA | NA |
| **Clinical outcomes** | Total complications (CD I-V) | NA | NA | NA | 9 (6.9%) | 14 (10.7%) | NA |
|  | Severe complications (CD =/> IIIa) | NA | NA | NA | NA | NA | NA |
|  | Postoperative pain score | NA | NA | NA | C30-Pain  STO22-Pain  Resting pain  Movement pain  Pain killer  PCA usage | Idem  Idem Idem Idem  Idem  Idem | *p*=0.169  *p*=0.789  *p*=0.614  *p*=0.421  *p*=0.824  *p*=0.524 |
|  | Length of stay (days; median [IQR]/mean +/- SD) | 10.2 +/- 11.1 | 11.1 +/- 11.2 | *p*=0.145 | NA | NA | NA |
| **Oncological outcomes** | Lymph node yield  (median [IQR]) | **19.6 +/ 13.0** | **17.4 +/- 11.6** | ***p*<0.001** | NA | NA | NA |
|  | Radicality of resection (R0) | 574 (91.7%) | 1133 (90.9%) | *p*=0.549 | NA | NA | NA |
| **Overall quality of life** | PRO/PROM (questionnaires) | NA | NA | NA | EORTC QLQ-C30 and STO22 | Idem | No difference in postoperative QoL, only better score on C30-cognitive functioning RG (*p*=0.005) |
| **Economic** | Costs | NA | NA | NA | NA | NA | NA |
| **Surgical experience** | Ergonomics | NA | NA | NA | NA | NA | NA |
| **Impact learning curve included? If yes, give a short description** | | No | | | This study was performed by surgeons who participated in the Korean Laparo-endoscopic Gastrointestinal Surgery Study Group, and the laparoscopic or robotic procedures were accordingly standardized in the multicenter. | | |

RG = Robot-assisted gastrectomy. LG = Laparoscopic gastrectomy. CD = Clavien-Dindo. SD = Standard deviation. NA = not applicable. PSM = propensity score matched. IQR = interquartile range.

|  | **Outcomes** | **Garbarino et al. (2021)** [39]  **(n=123)** | | | **Hikage et al. (2021)** [29]  **(n=684)** | | |
| --- | --- | --- | --- | --- | --- | --- | --- |
|  |  | **RG vs OG in the elderly (>70 years)** | | | **RG vs LG for cT1-2** | | |
|  |  | **Study type: PSM (1:1)** | | | **Study type: PSM (1:1)** | | |
|  |  | **Robot**  **(n=43)** | **Open**  **(n=43)** | **Difference between the groups (95% CI)** | **Robot**  **(n=342)** | **Laparoscopic**  **(n=342)** | **Difference between the groups (95% CI)** |
| **Extent of surgery** | Total gastrectomy (n (%))  Subtotal gastrectomy (n (%)) | 9 (21%)  34 (79%) | 12 (28%)  31 (72%) | *p*=0.654 | 25 (7%)  317 (93%) | 28 (8%) 314 (92%) | *p*=0.345 |
| **Survival** | Overall survival | 82.7% (1-yr OS)  52.3% (3-yr OS)  34.9% (5-yr OS) | 73.3% (1-yr OS)  41.8% (3-yr OS)  31.3% (5-yr OS) | *p*=0.263 | 96.4% (95%CI 92.9-98.2%)  (5-yr OS) | 94.8% (95%CI 89.9-97.4%) (5-yr OS) | *p*=0.532 |
|  | Disease free survival | 65.1% (1-yr OS)  51.6% (3-yr OS)  51.6% (5-yr OS) | 62.6% (1-yr OS)  42.4% (3-yr OS)  42.4% (5-yr OS) | *p*=0.474 | 95.2% (95%CI 91.7-97.3%)  (5-yr DFS) | 93.4% (95%CI 88.5-96.3%)  (5-yr DFS) | *p*=0.469 |
|  | 30-day mortality | 1 (2.3%) | 0 (0.0%) | *p*=1.000 | NA | NA | NA |
| **Intraoperative details** | Conversion to open (n (%)) | 6 (14.0%) | 0 (0.0%) | NA |  |  |  |
|  | Intraoperative complications (n (%)) | NA | NA | NA | NA | NA | NA |
| **Clinical outcomes** | Total complications (CD I-V) | 16 (37.2%) | 18 (41.9%) | *p*=0.662 | 45 (13.2%) | 63 (18.4%) | *p*=0.074 |
|  | Severe complications (CD =/> IIIa) | 3 (6.9%) | 7 (16.3%) | *p*=0.313 | NA | NA | NA |
|  | Postoperative pain score | **0.95 +/-0.7 (4-point VRS)** | **1.24 +/-0.7 (4-point VRS)** | ***p*=0.042** | NA | NA | NA |
|  | Length of stay (days; median [IQR]/ mean +/- SD) | 9 [7-90] | 9 [6-25] | *p*=0.685 | **8 (6-129)** | **9 (6-165)** | ***p*=0.041** |
| **Oncological outcomes** | Lymph node yield  (median [IQR]) | 22.1 +/- 8.4 | 22.5 +/- 12.8 | *p*=0.856 | 42 (16-99) | 40.5 (13-99) | *p*=0.842 |
|  | Radicality of resection (R0) | 41 (95.3%) | 37 (86.0%) | *p*=0.181 | NA | NA | NA |
| **Overall quality of life** | PRO/PROM (questionnaires) | NA | NA | NA | NA | NA | NA |
| **Economic** | Costs | NA | NA | NA | NA | NA | NA |
| **Surgical experience** | Ergonomics | NA | NA | NA | NA | NA | NA |
| **Impact learning curve included? If yes, give a short description** | | No | | | All RG procedures were performed by one of seven skilled surgeons who had received board-certification from the Japanese Society for Endoscopic Surgery and who completed a da Vinci surgery training program. They also performed or supervised all LG procedures in the present study. A da Vinci S, Si, or Xi Surgical System was used for RG. A detailed explanation about our RG procedure has been published elsewhere. | | |

RG = Robot-assisted gastrectomy. OG = Open gastrectomy. LG = laparoscopic gastrectomy. OS = overall survival. DFS = disease free survival. SD = standard deviation. NA = not applicable. PSM = propensity score matched. VRS = verbal rating scale. IQR = interquartile range.

|  | **Outcomes** | **Li et al. (2021)** [31]  **(n=816)** | | | **Nakauchi et al. (2021)** [36]  **(n=311)** | | |
| --- | --- | --- | --- | --- | --- | --- | --- |
|  |  | **RG vs LG** | | | **RG vs LG** | | |
|  |  | **Study type: PSM (1:1)** | | | **Study type: retrospective monocenter study** | | |
|  |  | **Robot (n=408)** | **Laparoscopic (n=408)** | **Difference between the groups (95% CI)** | **Robot**  **(n=190)** | **Laparoscopic**  **(n=121)** | **Difference between the groups (95% CI)** |
| **Extent of surgery** | Total gastrectomy (n (%))  Subtotal gastrectomy (n (%)) | 128 (31%)  280 (69%) | 151 (37%)  257 (63%) | *p*=0.104 | 61 (32%)  129 (68%) | 35 (29%)  86 (71%) | *p*=0.554 |
| **Survival** | Overall survival | 307 (76.7%)  (3-yr OS) | 297 (73.3%)  (3-yr OS) | *p*=0.246 | NA | NA | NA |
|  | Disease free survival | 305 (76.2%) (3-yr DFS) | 285 (70.1%) (3-yr DFS) | *p*=0.076 | 82.3%  (5-yr DSS) | 81.3%  (5-yr DSS) | *p*=0.873 |
|  | 30-day mortality | NA | NA | NA | 1 (0.5%) | 1 (0.8%) | *p*=0.628 |
| **Intraoperative details** | Conversion to open (n (%)) | NA | NA | NA | 49 (25.8%) | 48 (39.7%) | ***p*=0.010** |
|  | Intraoperative complications (n (%)) | NA | NA | NA | NA | NA | NA |
| **Clinical outcomes** | Total complications (CD I-V) | NA | NA | NA | 36 (18.9%) | 28 (23.1%) | *p*=0.373 |
|  | Severe complications (CD =/> IIIa) | NA | NA | NA | 11 (5.8%) | 16 (13.2%) | ***p*=0.023** |
|  | Postoperative pain score | NA | NA | NA | NA | NA | NA |
|  | Length of stay (days; median [IQR]/mean +/- SD) | NA | NA | NA | 5 [4-7] | 6 [5-7] | ***p*=0.045** |
| **Oncological outcomes** | Lymph node yield  (median [IQR]) | NA | NA | NA | 27 [20-38] | 22 [15-31] | ***p*<0.001** |
|  | Radicality of resection (R0) | NA | NA | NA | NA | NA | NA |
| **Overall quality of life** | PRO/PROM (questionnaires) | NA | NA | NA | NA | NA | NA |
| **Economic** | Costs | NA | NA | NA | NA | NA | NA |
| **Surgical experience** | Ergonomics | NA | NA | NA | NA | NA | NA |
| **Impact learning curve included? If yes, give a short description** | | ‘All surgeries were performed by 5 expert surgeons who had overcome the learning curve or RG and LG (surgeons have completed 15 cases of RG and 100 cases of LG based on previous research.’ | | | ‘We started performing robotic gastrectomy in 2012 after achieving our learning curve with more than 100 laparoscopic gastric procedures. Since then, the robotic approach has been applied in most cases eligible for a MIS approach.’ | | |

RG = robot-assisted gastrectomy. LG = laparoscopic gastrectomy. OS = overall survival. DFS = disease-free survival. DSS = disease specific survival. CD = Clavien-Dindo. NA = not applicable. SD = standard deviation. PSM = propensity score matched. IQR = interquartile range.

|  | **Outcomes** | **Nishi et al. (2021)** [40]  **(n=158)** | | | **Roh et al. (2021)** [37]  **(n=148)** | | |
| --- | --- | --- | --- | --- | --- | --- | --- |
|  |  | **RG vs LG** | | | **RTG vs LTG** | | |
|  |  | **Study type: PSM (1:1)** | | | **Study type: PSM (1:1)** | | |
|  |  | **Robot**  **(n=79)** | **Laparoscopic**  **(n=79)** | **Difference between the groups (95% CI)** | **Robot**  **(n=74)** | **Laparoscopic**  **(n=74)** | **Difference between the groups (95% CI)** |
| **Extent of surgery** | Total gastrectomy (n (%))  Subtotal gastrectomy (n (%)) | 26 (33%) 53 (67%) | 36 (46%) 43 (54%) | *p*=0.359 | 74 (100%)  0 (0%) | 74 (100%)  0 (0%) | NA |
| **Survival** | Overall survival | 91.72% (3-yr OS) | 83.39% (3-yr OS) | NS | 98.6% (3-yr OS) | 89.7% (3-yr OS) | *p*=0.144 |
|  | Disease free survival | 93.31%  (3-yr DFS) | 90.44%  (3-yr DFS) | NS | 97.3% (3-yr RFS) | 87.0%  (3-yr RFS) | *p*=0.167 |
|  | 30-day mortality | NA | NA | NA | 0 (0.0%) | 0 (0.0%) | NA |
| **Intraoperative details** | Conversion to open (n (%)) | NA | NA | NA | 0 (0.0%) | 1 (1.4%) | *p*>0.999 |
|  | Intraoperative complications (n (%)) | NA | NA | NA | 2 (2.7%) | 1 (1.4%) | *p*>0.999 |
| **Clinical outcomes** | Total complications (CD I-V) | NA | NA | NA | 23 (31.1%) | 23 (31.1%) | *p*=0.893 |
|  | Severe complications (CD =/> IIIa) | 2 (3%) | 4 (5%) | *p*=0.405 | 8 (10.8%) | 11 (14.9%) | NA |
|  | Postoperative pain score | NA | NA | NA | NA | NA | NA |
|  | Length of stay (days; median [IQR]/mean +/- SD) | 11.73 +/- 4.58 | 16.15 +/- 10.70 | ***p*<0.0001** | 9.0 +/- 15.0 | 9.2 +/- 5.2 | *p*=0.795 |
| **Oncological outcomes** | Lymph node yield  (median [IQR]) | 35.02 +/- 15.51 | 25.28 +/- 11.70 | ***p*<0.0001** | 43.1 +/- 14.9 | 46.0 +/- 14.4 | *p*=0.232 |
|  | Radicality of resection (R0) | NA | NA | NA | 74 (100%) | 74 (100%) | NA |
| **Overall quality of life** | PRO/PROM (questionnaires) | NA | NA | NA | NA | NA | NA |
| **Economic** | Costs | NA | NA | NA | NA | NA | NA |
| **Surgical experience** | Ergonomics | NA | NA | NA | NA | NA | NA |
| **Impact learning curve included? If yes, give a short description** | | ‘The present study has some limitations. The learning curve may affect the surgical outcome of both LG and RG. Multicenter RCT’s with larger sample sizes are warranted.’ | | | ‘In this study, textbook outcome was comparable between RTG and LTG groups. Because robotic and laparoscopic surgeries fall into the same domain of minimally invasive approaches, it may be a major obstacle for robotic surgery to offer significant surgical advantages compared to laparoscopic surgery. Moreover, because RTG has been used for proximal gastric cancer since 2009 in our institution, RTG included initial and early experiences, while LTG included experiences after the learning curve in this study. Therefore, it would be more difficult to show the difference between the two methods in terms of textbook outcome.’ | | |

RG = robot-assisted gastrectomy. LG = laparoscopic gastrectomy. RTG = robot-assisted total gastrectomy. LTG = laparoscopic total gastrectomy. NS = not significant. NA = not applicable. RFS = relapse-free survival. CD = Clavien-Dindo.
PSM = propensity score matched. IQR = interquartile range. RCT = Randomized Controlled Trial.

|  | **Outcomes** | **Shin et al. (2021)** [38]  **(n=778)** | | | **Tian et al. (2021)** [33]  **(n=912)** | | |
| --- | --- | --- | --- | --- | --- | --- | --- |
|  |  | **RG vs LG** | | | **RG vs LG** | | |
|  |  | **Study type: propensity score weighting** | | | **Study type: PSM (1:1)** | | |
|  |  | **Robot**  **(n=420)** | **Laparoscopic**  **(n=358)** | **Difference between the groups (95% CI)** | **Robot**  **(n=456)** | **Laparoscopic**  **(n=456)** | **Difference between the groups (95% CI)** |
| **Extent of surgery** | Total gastrectomy (n (%))  Subtotal gastrectomy (n (%)) | 72 (17%) 348 (83%) | 58 (16%) 300 (84%) | *p*=0.651 | 99 (22%)  357 (78%) | 108 (24%)  348 (76%) | *p*=0.477 |
| **Survival** | Overall survival | 93.2%  (95%CI 92.1-96.6)  (5-yr OS) | 94.2%  (95%CI 92.8-95.6)  (5-yr OS) | 95%CI 0.52-1.48, *p*=0.636 | 81.2% (3-yr OS) | 80.3% (3-yr OS) | *p*=0.648 |
|  | Disease free survival | 95.3% (95%CI 93.3-97.3)  (5-yr RFS) | 96.3%  (95%CI 95.1-97.5)  (5-yr RFS) | 95%CI 0.66-2.33, *p*=0.498 | 76.6%  (95%CI 31.20-32.83)  (3-yr RFS) | 77.0%  (95%CI 30.91-32.61)  (3-yr RFS) | *p*=0.951 |
|  | 30-day mortality | 1.0 (0.2%) | 0.7 (0.2%) | *p*=0.839 | 1 (0.2%) | 3 (0.6) | *p*=0.316 |
| **Intraoperative details** | Conversion to open (n (%)) | 0.0 (0.0%) | 0.6 (0.2%) | ***p*=0.020** | 3 (0.6%) | 7 (1.5%) | *p*=0.203 |
|  | Intraoperative complications (n (%)) | NA | NA | NA | NA | NA | NA |
| **Clinical outcomes** | Total complications (CD I-V) | 96.0 (22.8%) | 69.6 (19.4%) | *p*=0.340 | 80 (17.3%) | 80 (17.6%) | *p*=0.836 |
|  | Severe complications (CD =/> IIIa) | 24.0 (5.7%) | 16.1 (4.5%) | *p*=0.340 | 12 (2.6%) | 15 (3.3%) | *p*=0.836 |
|  | Postoperative pain score | NA | NA | NA | NA | NA | NA |
|  | Length of stay (days; median [IQR]/ mean +/- SD) | 7.30 +/- 6.44 | 6.44 +/- 3.26 | *p*=0.105 | 7.31 +/- 7.83 | 7.60 +/- 8.97 | *p*=0.218 |
| **Oncological outcomes** | Lymph node yield  (median [IQR]) | 38.75 +/- 13.51 | 37.40 +/- 13.16 | *p*=0.102 | 32.15 +/- 13.98 | 30.82 +/- 13.25 | ***p*=0.040** |
|  | Radicality of resection (R0) | NA | NA | NA | NA | NA | NA |
| **Overall quality of life** | PRO/PROM (questionnaires) | NA | NA | NA | NA | NA | NA |
| **Economic** | Costs | NA | NA | NA | $13.607 +/- 4.375 | $10.928 +/- 3.918 | ***p*<0.001** |
| **Surgical experience** | Ergonomics | NA | NA | NA | NA | NA | NA |
| **Impact learning curve included? If yes, give a short description** | | ‘LG was performed by 3 qualified surgeons who have had experience with more than 60 cases of gastrectomy by MIS. RG was performed by 1 qualified surgeon who has performed more than 60 cases of LG and also received training for robotic surgery that included a proctorship and animal training.’ | | | ‘LG was performed by professor ZYB’s surgery team, including three qualified surgeons who had an experience of more than 50 LGs. RG was performed by professor ZYB, who had performed more than 20 RGs and had received professional training for robotic surgery to obtain a surgery certificate.’ | | |

RG = robot-assisted gastrectomy. LG = laparoscopic gastrectomy. PSM = propensity score matched. OS = overall survival. RFS = recurrence free survival. NA = not applicable. CD = Clavien-Dindo. SD = standard deviation. MIS = minimally invasive surgery. IQR = interquartile range.

|  | **Outcomes** | **Gao et al. (2022)** [42] **(n=1164)** | | | **Hikage et al. (2022)** [35] **(n=1306)** | | |
| --- | --- | --- | --- | --- | --- | --- | --- |
|  |  | **RADG vs. LADG** | | | **RG vs LG** | | |
|  |  | **Study type: PSM** | | | **Study type: retrospective cohort study, patients with high visceral fat** | | |
|  |  | **RADG**  **(n=410)** | **LADG**  **(n=410)** | **Difference between the groups** | **RG**  **(n=394)** | **LG**  **(n=882)** | **Differences between the groups** |
| **Extent of surgery** | Total gastrectomy (n (%))  Subtotal gastrectomy (n (%)) | 0 (0%)  410 (100%) | 0 (0%)  410 (100%) | NA | 38 (10%) 356 (90%) | 58 (7%)  824 (93%) | NA |
| **Survival** | Overall survival | 75.5% (3-yrs) | 73.1% (3-yrs) | *p*=0.471 | 96.5% (95%CI 89.5-98.9) (5-yrs) | 90.1% (95%CI 85.3-93.4%) (5-yrs) | ***p*=0.045** |
|  | Disease free survival | 72.9% (3-yrs) | 71.4% (3-yrs) | *p*=0.763 | NA | NA | NA |
|  | 30-day mortality | 3 (0.6%) | 4 (1.0%) | *p*=0.704 | NA | NA | NA |
| **Intraoperative details** | Conversion to open (n (%)) | 3 (0.7%) | 6 (1.5%) | *p*=0.315 | NA | NA | NA |
|  | Intraoperative complications (n (%)) | NA | NA | NA | NA | NA | NA |
| **Clinical outcomes** | Total complications (CD I-V) | 56 (13.7%) | 68 (16.6%) | *p*=0.242 | NA | NA | NA |
|  | Severe complications (CD =/> IIIa) | 11 (2.7%) | 15 (3.7%) | *p*=0.425 | 4 (2.7%) high fat | 30 (8.2%) | *p*=0.019 |
|  | Postoperative pain score | NA | NA | NA | NA | NA | NA |
|  | Length of stay (days; median [IQR]/ mean +/- SD) | 9.0 +/- 3.9 | 9.1 +/- 3.5 | *p*=0.371 | 8 (6-129) high fat | 9 (6-70) | ***p*=0.013** |
| **Oncological outcomes** | Lymph node yield  (median [IQR]) | 31.4 +/- 12.1 | 29.4 +/- 12.3 | ***p*=0.015** | 36 | 37 | *p*=0.488 |
|  | Radicality of resection (R0) | NA | NA | NA | NA | NA | NA |
| **Overall quality of life** | PRO/PROM (questionnaires) | NA | NA | NA | NA | NA | NA |
| **Economic** | Costs | 13.608 +/- 4326 dollars | 10.925 +/- 3925 dollars | ***p*<0.001** | NA | NA | **NA** |
| **Surgical experience** | Ergonomics | NA | NA | NA | NA | NA | NA |
| **Impact learning curve included? If yes, give a short description** | | No | | | RG was introduced for in-house phase II studies in January 2012. All the RG procedures were performed by one of seven experienced surgeons who were board-qualified by the Japanese Society for Endoscopic Surgery and had completed a training program for da Vinci Surgery. | | |

RADG = Robot-assisted distal gastrectomy. LADG = Laparoscopic-assisted distal gastrectomy. RG = robot-assisted gastrectomy. LG = laparoscopic gastrectomy. OS = overall survival. DFS = disease free survival. NA = not applicable.
CD = Clavien-Dindo. SD = standard deviation. IQR = interquartile range. PSM = propensity score matched.

|  | **Outcomes** | **Li et al. (2022)** [30] **(n=1327)** | | |
| --- | --- | --- | --- | --- |
|  |  | **RG vs LG** | | |
|  |  | **Study type: PSM 1:3** | | |
|  |  | **RG**  **(n=221)** | **LG**  **(n=663)** | **Differences between the groups** |
| **Extent of surgery** | Total gastrectomy (n (%))  Subtotal gastrectomy (n (%)) | 94 (43%)  127 (58%) | 308 (47%)  355 (54%) | *p*=0.311 |
| **Survival** | Overall survival | 81.0% (3-yrs) OS | 79.3% (3-yrs) OS | *p*=0.516 |
|  | Disease free survival | 78.7% (3-yrs) RFS | 75.6% (3-yrs) RFS | *p*=0.600 |
|  | 30-day mortality | 36 (16.3%) | 118 (17.8%) | *p*=0.609 |
| **Intraoperative details** | Conversion to open (n (%)) | NA | NA | NA |
|  | Intraoperative complications (n (%)) | NA | NA | NA |
| **Clinical outcomes** | Total complications (CD I-V) | 36 (16.3%) | 118 (17.8%) | *p*=0.449 |
|  | Severe complications (CD =/> IIIa) | 5 (2.3%) | 9 (1.4%) |  |
|  | Postoperative pain score | NA | NA | NA |
|  | Length of stay  (days; median [IQR]/mean +/- SD) | 9.6 +/- 4.1 | 9.4 +/- 3.5 | *p*=0.486 |
| **Oncological outcomes** | Lymph node yield  (median [IQR]) | 40.2 +/- 15 (RTG) 35.0 +/- 11.9 (RDG) | 42.1 +/- 15.1 36.9 +/- 13.8 | *p*=0.288 *p*=0.138 |
|  | Radicality of resection (R0) | NA | NA | NA |
| **Overall quality of life** | PRO/PROM (questionnaires) | NA | NA | NA |
| **Economic** | Costs | 15262 dollar | 10945 dollar | ***p*<0.001** |
| **Surgical experience** | Ergonomics | NA |  |  |
| **Impact learning curve included? If yes, give a short description** | | No | | |

RG = robot-assisted gastrectomy. LG = laparoscopic gastrectomy. OS = overall survival. RFS = recurrence free survival. NA = not applicable. CD = Clavien-Dindo.
PSM = propensity score matched. SD = standard deviation. IQR = interquartile range.

|  | **Outcomes** | **Suda et al. (2022)** [44] **(n=1127)** | | | **Kamarajah (2022)** [32]  **(n=30.324)** | | | |
| --- | --- | --- | --- | --- | --- | --- | --- | --- |
|  |  | **RG vs LG in stage I/II gastric cancer** | | | **RAMIG vs LAMIG vs OG** | | | |
|  |  | **Study type: multi-institutional retrospective study, PSM** | | | **Study type: Population-based cohort study** | | | |
|  |  | **RG**  **(n=326)** | **LG**  **(n=752)** | **Differences between groups [95%CI]** | **RAMIG**  **(n=1721)** | **LAMIG  (n=6375)** | **OG (n=22.228)** | **Difference between the groups (95% CI)** |
| **Extent of surgery** | Total gastrectomy (n (%))  Subtotal gastrectomy (n (%))  Unknown | 74 (23%)  252 (77%) - | 162 (22%)  590 (78%) - | NA | 492 (29%)  1193 (69%) 36 (2%) | 1740 (27%) 4532 (71%) 103 (2%) | 6853 (31%) 14992 (67%) 383 (2%) | NA |
| **Survival** | Overall survival | 96.3% (3-yrs) | 89.6% (3-yrs) | ***p*=0.009 for RG** | Median 66.4 (58.0-84.9) (5-yrs) | 63.6 (58.5-68.5)  (5-yrs) | 42.5 (40.8-44.1)  (5-yrs) | ***p*<0.001 (LAMIG vs. OG)**  ***p*=0.006 (RG vs. OG**) *p*=0.800 (RG vs. LG) |
|  | Disease free survival | 92.3% (3-yrs RFS) | 87.2% (RFS) | *p*=0.073 | NA | NA | NA | NA |
|  | 30-day mortality | 0 (0%) in hospital mortality | 3.7 (0.3%) | ***p*<0.001 for RG** | 38 (2.2%) | 172 (2.7%) | 969 (4.4%) | ***p*<0.001** |
| **Intraoperative details** | Conversion to open (n (%)) | 8.4 (0.8%) | 1.1 (0.1%) | *p*=0.164 | NA | *NA* | *NA* | *NA* |
|  | Intraoperative complications (n (%)) | NA | NA | NA | NA | NA | NA | NA |
| **Clinical outcomes** | Total complications (CD I-V) | NA | NA | NA | **34%** | **35%** | **36%** | *p*=0.319 |
|  | Severe complications (CD =/> IIIa) | 40.8 (3.7%) | 53.8 (5%) | *p*=0.632 | 65 (3.8%) | 237 (3.7%) | 822 (3.7%) | *p*=0.319 |
|  | Postoperative pain score | NA | NA | NA | NA | NA | NA | NA |
|  | Length of stay (days; median [IQR]/ mean +/- SD) | 9 (6-62) | 12 (6-334) | − 4.4 [− 6.8, 2.0]  ***p*<0.001** | ≤21 days (8.3%) >21 days (91.7%) | ≤21 days (8.4%) >21 days (91.6%) | ≤21 days (10%)  >21 days (90%) | ***p*<0.001** |
| **Oncological outcomes** | Lymph node yield  (median [IQR]) | 40 (10-103) | 40 (8-115) | *p*=0.285 | >15 63% | **>**15 (58%) | **>15 (53%)** | ***p*<0.001 (whole)**  ***p*=0.0001 (RAMIG vs LAMIG)** |
|  | Radicality of resection (R0) | NA | NA | NA | 96.2% | 94.5% | 93.4% | ***p*<0.001 (whole)**  ***p*=0.008 (RG vs LG)** |
| **Overall quality of life** | PRO/PROM (questionnaires) | NA | NA | NA | NA | NA | NA | NA |
| **Economic** | Costs |  |  |  | NA | NA | NA | NA |
|  | - Medical costs  (median with ranges) | 1.800.084 (1.530.170 -  5.173.706) | 1.633.222 (1.139.526 -  11.781.742) | 242.028 [80.616-  403.440] ***p*=0.003 for LG** | Most patients undergoing OG were not insured! ***p*<0.001** | | | |
|  | - Surgical costs | 1.063.800 (950.000 -  1.158.970) | 823.070 (585.000 -  1.431.910) | 217.139 [201.407 -  232.871] ***p*<0.001 for LG** |  |  |  |  |
| **Surgical experience** | Ergonomics | NA | NA | NA | NA | NA | NA | NA |
| **Impact learning curve included? If yes, give a short description** | | To control for surgeon volume, an operating surgeon who had performed ≥ 100 LGs before any of the patients enrolled in the LG group had undergone surgery was defined as an expert surgeon in the LG group. Likewise, we recognized any RG surgeon who was able to perform LG expert procedures using the surgical robot as an RG expert, and an LG expert who had performed ≥40 RGs before any of the patients enrolled in the RG group had undergone surgery was defined as an expert surgeon in the RG group, considering the learning curve for RG among experienced LG surgeons. All procedures were performed or supervised by an expert surgeon. | | | NA | | | |

RG = robot-assisted gastrectomy. LG = laparoscopic gastrectomy. RAMIG = robot-assisted minimally invasive gastrectomy. LAMIG = laparoscopic-assisted minimally invasive gastrectomy. OG = open gastrectomy. RFS = recurrence free survival.
NA = not applicable. CD = Clavien-Dindo. SD = standard deviation. PSM = propensity score matched.

|  | **Outcomes** | **Li et al. (2023)** [43] **(n=5402)** | | |
| --- | --- | --- | --- | --- |
|  |  | **RG vs LG** | | |
|  |  | **Study type: retrospective multicenter PSM cohort study (1:1), prospective database** | | |
|  |  | **RG**  **(n=1776)** | **LG**  **(n=1776)** | **Differences between the groups** |
| **Extent of surgery** | Total gastrectomy (n (%))  Subtotal gastrectomy (n (%)) | 495 (28%)  1281 (72%) | 499 (28%)  1277 (72%) | *p*=0.947 |
| **Survival** | Overall survival | 83.5% (3-yrs)  80.8% (5-yrs) | 82.2% (3-yrs)  79.5% (5-yrs) | *p*=0.240  *p*=0.213 |
|  | Disease free survival | 82.6% (3-yrs)  79.8% (5-yrs) | 81.3% (3-yrs)  78.5% (5-yrs) | *p*=0.227  *p*=0.205 |
|  | 30-day mortality | 3 (3.2%) in hospital mortality | 2 (0.1%) | *p*=1.000 |
| **Intraoperative details** | Conversion to open (n (%)) | 21 (1.2%) | 29 (1.6%) | *p*=0.255 |
|  | Intraoperative complications (n (%)) | 118 (6.6%) are surgical complications | 152 (8.6%) | ***p*=0.031** |
| **Clinical outcomes** | Total complications (CD I-V) | 12.6% | 15.2% | ***p*=0.023** |
|  | Severe complications (CD =/> IIIa) | 2.5% | 2.9% | *p*=0.408 |
|  | Postoperative pain score | NA | NA | NA |
|  | Length of stay (days; median [IQR]/ mean +/- SD) | 9.2 +/- 3.9 | 9.3 +/- 3.5 | *p*=0.371 |
| **Oncological outcomes** | Lymph node yield  (median [IQR]) | 32.5 | 30.7 | ***p*<0.001 for RG** |
|  | Radicality of resection (R0) | NA | NA | NA |
| **Overall quality of life** | PRO/PROM (questionnaires) | NA | NA | NA |
| **Economic** | Costs | $14.185 +/- 4.892 | $10.637 +/- 4.398 | ***p<*0.001 for LG** |
| **Surgical experience** | Ergonomics | NA | NA | **NA** |
| **Impact learning curve included? If yes, give a short description** | | RGs and LGs were performed by 11 qualified surgeons who had completed >100 LGs and have received standardized training for RGs | | |

RG = robot-assisted gastrectomy. LG = laparoscopic gastrectomy. NA = not applicable. CD = Clavien-Dindo. SD = standard deviation. PSM = propensity score matched.
